# The impact of gestational weight gain on fetal and neonatal outcomes: the Araraquara Cohort Study

**DOI:** 10.1186/s12884-024-06523-x

**Published:** 2024-04-25

**Authors:** Audêncio Victor, Laísla de França da Silva Teles, Isabel Oliveira Aires, Leticia Falcão de Carvalho, Liania A. Luzia, Rinaldo Artes, Patrícia H. Rondó

**Affiliations:** 1 Public Health Postgraduate Program, School of Public Health, University of São Paulo, São Paulo, SP Brazil; 2 Nutrition Department, School of Public Health, University of São Paulo, São Paulo, SP Brazil; 3 Insper - Institute of Education and Research, São Paulo, Brazil; 4 Faculdade de Saúde Pública- USP, Avenida Doutor Arnaldo, 715 – São Paulo, São Paulo, Brazil

**Keywords:** Gestational weight gain, Pregnancy, Fetal outcomes, Neonatal outcomes, Cohort study, Intrauterine growth restriction

## Abstract

**Background:**

Gestational weight gain (GWG) is an important indicator for monitoring maternal and fetal health. Objective: To evaluate the effect of GWG outside the recommendations of the Institute of Medicine (IOM) on fetal and neonatal outcomes.

**Study design:**

A prospective cohort study with 1642 pregnant women selected from 2017 to 2023, with gestational age ≤ 18 weeks and followed until delivery in the city of Araraquara, Southeast Brazil. The relationship between IOM-recommended GWG and fetal outcomes (abdominal subcutaneous tissue thickness, arm and thigh subcutaneous tissue area and intrauterine growth restriction) and neonatal outcomes (percentage of fat mass, fat-free mass, birth weight and length, ponderal index, weight adequateness for gestational age by the Intergrowth curve, prematurity, and Apgar score) were investigated. Generalized Estimating Equations were used.

**Results:**

GWG below the IOM recommendations was associated with increased risks of intrauterine growth restriction (IUGR) (aOR 1.61; 95% CI: 1.14–2.27), low birth weight (aOR 2.44; 95% CI: 1.85–3.21), and prematurity (aOR 2.35; 95% CI: 1.81–3.05), and lower chance of being Large for Gestational Age (LGA) (aOR 0.38; 95% CI: 0.28–0.54), with smaller arm subcutaneous tissue area (AST) (-7.99 g; 95% CI: -8.97 to -7.02), birth length (-0.76 cm; 95% CI: -1.03 to -0.49), and neonatal fat mass percentage (-0.85%; 95% CI: -1.12 to -0.58). Conversely, exceeding GWG guidelines increased the likelihood of LGA (aOR 1.53; 95% CI: 1.20–1.96), with lower 5th-minute Apgar score (aOR 0.42; 95% CI: 0.20–0.87), and increased birth weight (90.14 g; 95% CI: 53.30 to 126.99).

**Conclusion:**

Adherence to GWG recommendations is crucial, with deviations negatively impacting fetal health. Effective weight control strategies are imperative.

**Supplementary Information:**

The online version contains supplementary material available at 10.1186/s12884-024-06523-x.

## Introduction

Pregnancy is a critical window in maternal and child health, where gestational weight gain (GWG) emerges as a determinant factor for fetal well-being and development. Monitoring GWG is essential, serving as a prognostic marker for the health of the pregnant woman and the conceptus [1–5].

Guidelines for GWG have changed over time, reflecting the understanding of its importance. In the 1950s, the recommendation was for a gain of 10–14 lb, aiming to prevent complications such as pre-eclampsia (Ferguson et al., 1950). The following decade recognized the positive association between higher GWG and birth weight [5]. In 1990, the Institute of Medicine (IOM) of the USA established specific recommendations, with the most recent update in 2019, emphasizing the importance of not exceeding GWG targets to avoid obesity-related complications [6]. To mitigate adverse impacts on maternal and fetal health, the IOM in 2009 outlined guidelines suggesting a total weight gain of 11 to 16 kg for women of normal weight, 7 to 11 kg for overweight women, and 5 to 9 kg for obese women [7].

Excessive or insufficient GWG can increase the risk of negative outcomes for both the mother and the conceptus [8–10]. Research indicates that GWG above IOM recommendations is associated with an increased risk of metabolic complications, hypertension, gestational diabetes, cesarean section, postpartum weight retention, macrosomia, childhood obesity, and cardiometabolic outcomes in childhood [8–10]. Conversely, GWG below the recommendations is linked to an elevated risk of intrauterine growth restriction (IUGR), low birth weight (LBW), preterm birth, perinatal mortality, and a higher incidence of newborns small for gestational age (SGA) [11–13]. Furthermore, studies indicate that GWG within the IOM-recommended ranges is associated with a lower incidence of LBW and better health outcomes [14].

The possibility of mitigating the risks of gestational complications and adverse fetal outcomes in obese pregnant women by limiting GWG is an area of growing interest [15, 16]. The literature has highlighted the importance of adjusting GWG in various interventions, including diet, physical activity, the use of probiotics, and psychological and behavioral approaches [17].

In Brazil, although there are no standardized recommendations for GWG based on Brazilian population data, new guidelines for GWG have been proposed based on national GWG curves. Thus, the IOM recommendations, widely used to classify GWG, still constitute a viable alternative. Given this context, the objective of this study is to evaluate whether pregnant women with GWG outside the IOM-recommended range present adverse fetal and neonatal outcomes compared to those whose GWG is considered adequate, aiming to provide support for clinical practice and contribute to the development of effective intervention strategies that promote a healthy pregnancy and minimize risks to the mother and respective conceptus.

## Materials and methods

### Study design and participants

**Fig. 1:**
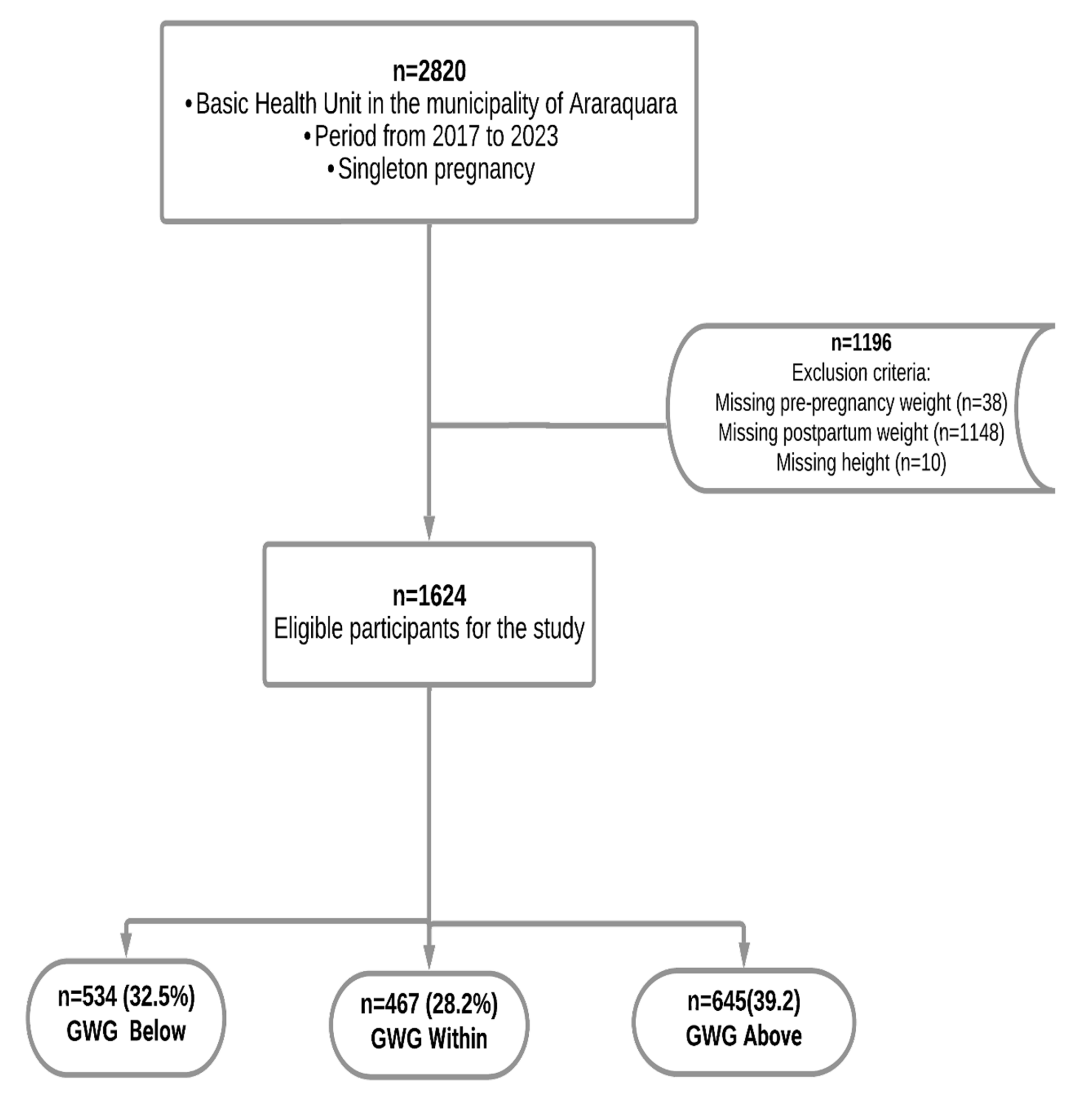
Selection of study population in a cohort study in Araraquara

A population-based prospective epidemiological cohort study, part of the ongoing “Araraquara Cohort Study”, was conducted. The sample included pregnant women with gestational age ≤ 18 weeks, recruited from Health Units in Araraquara, São Paulo, Brazil, and monitored quarterly until delivery from 2017 to 2023. Women with multiple pregnancies or who experienced miscarriage were excluded. Pregnant women lacking information on height, pre-pregnancy weight, and weight at delivery were also excluded (Fig. 1**).**

### Main exposure: GWG

GWG was calculated from the difference between weight at delivery and pre-pregnancy weight, with classifications according to IOM recommendations. Pre-pregnancy BMI was determined from measurements taken at the first assessment. For women with gestational age < 13 weeks, current weight was used; for those ≥ 13 weeks, weight recorded at the first prenatal visit was used. Nutritional status classification followed WHO guidelines: underweight (BMI < 18.5 kg/m²), normal weight (18.5–24.9 kg/m²), overweight (25–29.9 kg/m²), or obesity (≥ 30 kg/m²) [18].

### Fetal outcomes

Fetal variables were assessed longitudinally throughout the three trimesters of pregnancy, including abdominal subcutaneous tissue thickness (AST), arm subcutaneous tissue area (ASA), thigh subcutaneous tissue area (TSA), estimated fetal weight (EFW), and the diagnosis of IUGR, the latter based on the comparison of EFW with the fetal curve of the Intergrowth-21st Project. The diagnosis of IUGR was considered when the EFW was below the 10th percentile of the fetal curve [19, 20].

### Neonatal outcomes

The neonatal outcomes evaluated were the percentage of fat mass (%FM), percentage of fat-free mass (%FFM), LBW, birth weight (g), birth length (cm), ponderal index (length/weight³), gestational age at birth (preterm and term), and adequateness of birth weight according to the Intergrowth-21st - large for gestational age (LGA) (> 90th percentile), SGA (< 10th percentile), and adequate for gestational age (AGA) (between the 10th and 90th percentiles) [20]. The body composition of the neonates, including %FM and %FFM, was assessed using air displacement plethysmography.

### Maternal characteristics (study covariates)

Various factors were considered as covariates, including socioeconomic and demographic characteristics: age (years), education (years), number of people per room, number of previous delivery, parity, per capita income in reais (1 US$ = 4.9 R$), race (white or non-white), and marital status (married/in a stable union, single/separated/widowed). Lifestyle habits such as physical activity, smoking, and alcohol consumption were also considered, as well as morbidity (diabetes, hypertension, urinary tract infection). The anthropometry of pregnant women (pre-pregnancy weight, current BMI), gestational age, glycemic profile (fasting glucose, insulin, HOMA, glycated hemoglobin), high-sensitivity C-reactive protein (hs-CRP), hemoglobin, and lipid profile (triglycerides, total cholesterol, and fractions).

### Statistical analysis

A directed acyclic graph (DAG) was created to visually represent the theoretical model and clarify the relationships between variables related to GWG, as well as fetal and neonatal outcomes. (Figure S1). The DAG was constructed using DAGitty software (version 3.0; Nijmegen, GE, Netherlands) [21] Graphical criteria were applied for the selection of adjusted covariates, aiming to minimize bias in variable selection [22, 23].

Descriptive statistics was used for data analysis, and the Shapiro-Wilk test was utilized to check the normality of continuous variables. Non-normal continuous variables were presented as median and interquartile range (IQR), and categorical ones, in frequencies and percentages (n (%)). Bivariate analyses were conducted to examine associations between independent and dependent variables. The Kruskal-Wallis test was used for continuous variables and the chi-square or Fisher’s exact test for categorical ones. Generalized Linear Models were applied to associate GWG with neonatal outcomes (linear regression for continuous and logistic for binary). For fetal outcomes, longitudinal analysis with Generalized Estimating Equation (GEE) was used, considering the correlation between repeated observations of each pregnant woman [24–26]. Statistical analyses investigated associations between GWG and the outcomes of interest, considering the different GWG recommendations. The results were presented in measures of association (OR and Beta coefficient (β)), with statistical significance indicated by *P* < 0.05 and 95% confidence intervals. All analyses were performed using R software (version 4.1.0, R Foundation for Statistical Computing, Vienna, Austria).

## Results

### Maternal characteristics

Table 1. In this study, the association between maternal characteristics and GWG was evaluated in 1642 Brazilian women. The median age of the women was 27 years (IQR: 22.1–31.8), and the median years of education were 11 years (IQR: 10–11). The majority were in a stable relationship (*n* = 1440, 87.7%), with 54.1% (*n* = 889) identifying as non-white. The per capita income was R$ 667 (IQR: 409–1000), and the average number of previous delivery was 1 (IQR: 1–2).

**Table 1:** Maternal characteristics associated with GWG in three trimesters and its relationship with IOM recommendations

Charateristics	1st trimester	2st trimestre	3rd trimestre
Age (years )	27 (22.1–31.8)		
Maternal Education (years )	11(10–11)		
Number of Previous delivery †	1 (1–2)		
Number of people per room	0.6 (0.5–0.8)		
Per Capita Income in Brazilian Real (R$) †	667 (409–1000)		
Race			
White	753 (45.9)		
Non-white	889 (54.1)		
Marital Status, n(%)			
Married or in a stable relationship	1440 (87.7)		
Single, separated, or widowed	202 (12.3)		
Pre-gestational BMI (kg /m²) †	25.7 (22.2–30.4)		
Gestational BMI (kg /m²) †	25.8 (22.2–30.2)	27.4 (24.0-31.5)	28.8 (25.6–32.9)
Gestational Age (weeks) †	13.6 (12.3–15.3)	23.6 (22.1–25.1)	33(31.7–34.1)
Physical Activity			
Adequate	181 (11)	143 1 (8.7)	181 (11)
Inadequate	578 (35.2)	578 (35.2)	578 (35.2)
Smoking †			
No	1513 (92.1)	1422 (86.6)	1308 (79.7)
Yes	129 (7.0)	97 (5.9)	79 (4.8)
Alcohol Consumption			
No	1309 (79.7)	1384 (84.3)	1288 (78.4)
Yes	333 (20.3)	135 (8.2))	99 (6.0)
Diabetes, n(%) †			
No	1560 (95.0)	1395 (85.0)	1233 (75.1)
Yes	82 (5.0)	123 (7.5)	159 (9.7)
Hypertension			
No	1526 (92.9)	1405 (85.6)	1281 (78.0)
Yes	116 (7.1)	113 (6.9)	111 (6.8)
Urinary Tract Infection			
No	1449 (88.2)	1414 (86.1)	1306 (79.5)
Yes	193 (11.8)	104 (6.3)	86 (5.2)
Cervicitis/Vaginitis			
No	1531 (93.2)	1424 (86.7)	1322 (80.5)
Yes	111 (6.8)	94 (5.7)	70 (4.3)
hs-CRP (ng/mL)	6.0 (3.1–11.6)	6.1 (3.4–11.6) †	5 (3–10) †
Hemoglobin (g/dL) †	12.5 (11.9–13.1)	11.8 (11.3–12.4)	11.8 (11.3–12.4)
Glycated Hemoglobin %	5.1 (4.9–5.3)	4.9 (4.6–5.1)	5 (5–5)
Fasting Insulin (uUI/mL) †	7.0 (5–11)	8.0 (6–12)	10 (7–14)
Cholesterol (mg/dL)	173 (151–197)	210 (184–239)	226 (195–258)
HDL-c (mg/dL)	56.0 (48–64.0) †	61.0 (53–71) †	58 (50–68)
LDL-c (mg/dL)	95.0 (77–113)	116.0 (95.5–139)	127 (101–156)
Triglycerides (mg/dL)	104 (81–134)	146 (113–185)	186 (147–230) †

The data is presented as number (percentage) and median with interquartile range (25th percentile − 75th percentile)

† Statistical differences observed between gestational weight gain groups, tested with: Kruskal-Wallis test for continuous variables and χ2 test, Fisher’s test for categorical variables

Abbreviations: BMI: body mass index; LDL-c: low-density lipoprotein cholesterol; HDL-c: high-density lipoprotein cholesterol. 1 Brazilian Real (R$) equals approximately 4.9 US dollars (US$)

The median gestational BMI increased from 25.8 kg/m² (IQR: 22.2–30.2) in the first trimester to 28.8 kg/m² (IQR: 25.6–32.9) in the third trimester. Adequate physical activity was maintained by 11% of the women throughout all trimesters (*n* = 181). Smoking decreased from 7.9% (*n* = 129) in the first trimester to 4.8% (*n* = 79) in the third, and alcohol consumption from 20.3% (*n* = 333) to 6.0% (*n* = 99). Gestational diabetes increased from 5.0% (*n* = 82) to 9.7% (*n* = 159), showing an association with GWG.

Fasting insulin levels rose from 7.0 uIU/mL (IQR: 5–11) to 10 uIU/mL (IQR: 7–14) throughout pregnancy. Total cholesterol increased from 173 mg/dL (IQR: 151–197) to 226 mg/dL (IQR: 195–258), LDL-c from 95.0 mg/dL (IQR: 77–113) to 127 mg/dL (IQR: 101–156), and triglycerides from 104 mg/dL (IQR: 81–134) to 186 mg/dL (IQR: 147–230), all associated with GWG. HDL-c varied from 56 mg/dL (IQR: 48–64.0) in the first period to 58 mg/dL (IQR: 50–68) in the third.

### Neonatal and fetal characteristics

As shown in Table 2, fetal and neonatal outcomes were assessed in relation to GWG over the three periods of gestation. There was an increase in the AST, from 2.8 mm (IQR: 2.5–3.1) in the first period to 3.8 mm (IQR: 3.4–4.4) in the second trimester. The AST also showed growth, from 3.0 mm² (IQR: 2.4–3.6) to 6.6 mm² (IQR: 5.7–7.8), as did the TST, which increased from 5.4 mm² (IQR: 4.4–6.7) to 13.5 mm² (IQR: 11.4–15.9). The EFW increased from 171 g (IQR: 145–212) in the first period to 616 g (IQR: 486–776) in the second, reaching 2064 g (IQR: 1814–2341) in the third trimester. The prevalence of IUGR decreased from 16.3% (*n* = 268) to 1.5% (*n* = 23) from the first to the second period.

**Table 2:** Fetal and neonatal outcomes in the three trimesters according to GWG

Outcomes		1st trimester	2st trimester	3rd trimester
Fetal	Abdominal subcutaneous tissue thickness (TSA)		2.8 (2.5–3.1)	3.8 (3.4–4.4)
	Arm subcutaneous tissue area (TSB)		3.0 (2.4–3.6)	6.6 (5.7–7.8)
	Thigh subcutaneous tissue area (TSC)		5.4 (4.4–6.7)	13.5 (11.4–15.9)
	Estimated fetal weight in grams †	171 (145–212)	616 (486–776)	2064 (1814–2341)
	Intrauterine growth restriction†			
	No		918 (55.9)	1346 (82.0)
	Yes		268 (16.3)	23 (1.5)
Neonatal (a)	% Fat mass (%FM) †			21 (17–24)
	% Fat-free mass (%FFM) †			79 (76–83
	Prematurity †			
	No			1474 (89.7)
	Yes			154 (9.3)
	Apgar score at 5 minutes			
	No			21 (1.3)
	Yes			1594 (97.1)
	Birth length (cm) †			49.0 (47–50)
	Birth weight (g) †			3222 (2895–3520
	Adequateness of birth weight (Intergrowth) †			
	SGA			1155 (70.3)
	AGA			144 (8.8)
	LGA			137 (8.3)
	Low birth weight†			
	No			1488 (90.6)
	Yes			142 (8.6)

The data is presented as number (percentage) and median with interquartile range (25th percentile − 75th percentile)

† Statistical differences observed between gestational weight gain groups, tested with: Kruskal-Wallis test for continuous variables and χ2 test, Fisher’s test for categorical variables. (a) Variables measured at birth

In neonatal outcomes, the %FM at birth was 21% (IQR: 17–24), and the %FFM was 79% (IQR: 76–83). The prevalence of prematurity was 9.3% (*n* = 154), while most neonates were born at term (89.7%, *n* = 1474). The Apgar score at the 5th minute was satisfactory (≥ 7) in 97.1% of cases (*n* = 1594). The length at birth was 49.0 cm (IQR: 47–50), and the birth weight was 3222 g (IQR: 2895–3520). The adequateness of birth weight, according to the Intergrowth curve, showed 70.3% (*n* = 1155) as AGA, 8.8% (*n* = 144) as SGA, and 8.3% (*n* = 137) as LGA. The incidence of LBW was 8.6% (*n* = 142), with most neonates presenting with an adequate weight 90.6% (*n* = 1488).

### Association between GWG and fetal and neonatal outcomes

Tables 3 and 4 present the adjusted associations between GWG and fetal and neonatal outcomes. The results indicate that a GWG below the recommended range was associated with increased risks of IUGR (aOR 1.61; 95% CI: 1.14–2.27), LBW (aOR 2.44; 95% CI: 1.85–3.21), and prematurity (aOR 2.35; 95% CI: 1.81–3.05), and a lower chance of being LGA (aOR 0.38; 95% CI: 0.28–0.54), AST (-7.99 g; 95% CI: -8.97 to -7.02), birth length (-0.76 cm; 95% CI: -1.03 to -0.49), and neonatal fat mass percentage (-0.85%; 95% CI: -1.12 to -0.58). Whereas a GWG above the recommended range resulted in a higher likelihood of neonates being LGA (aOR 1.53; 95% CI: 1.20–1.96), with a lower Apgar score at the 5th minute (aOR 0.42; 95% CI: 0.20–0.87), and a higher birth weight (90.14 g; 95% CI: 53.30 to 126.99).

**Table 3:** Unadjusted and adjusted analysis to assess adverse fetal and neonatal outcomes (Categorical) associated with GWG

Outcomes	Gestacional weight gain (IOM-2019)			
	Below	Above	Below	Above
	Crude OR (IC95%)	Crude OR (IC95%)	Adjusted OR (IC95%)	Adjusted OR (IC95%)
Intrauterine growth restriction (FGR)	**1.53 (1.11–2.10)**	1.27 (0.92–1.74)	**1.61 (1.14–2.27)**	1.26 (0.89–1.78)
Prematurity	**2.34 (1.81–3.02)**	1.19 (0.91–1.57)	**2.35 (1.81–3.05)**	1.24 (0.94–1.64)
Apgar score at 5 minutes	0.58 (0.26–1.28)	**0.34 (0.16–0.71)**	0.52 (0.24–1.16)	**0.42 (0.20–0.87)**
Small for gestational age (SGA)	1.18 (0.92–1.50)	0.78 (0.60–1.01)	1.18 (0.92–1.53)	0.77 (0.59-1.00)
Large for gestational age (LGA)	**0.43 (0.32–0.60)**	**1.54 (1.21–1.95)**	**0.38 (0.28–0.54)**	**1.53 (1.20–1.96)**
Low birth weight (LBW)	**2.32 (1.78–3.03)**	1.17 (0.88–1.55)	**2.44 (1.85–3.21)**	1.21 (0.91–1.62)

The association between each fetal or neonatal outcome and GWG. Analyses adjusted for: Maternal age; marital status; race/color; mothers’ education in years of study; BMI; gestational age in weeks; parity; number of rooms per person; lactose consumption; smoking; diabetes; serum levels of hemoglobin; glycated hemoglobin; cholesterol; triglycerides; HDL; LDL. Estimated odds ratios (OR) using a generalized estimating equation. OR, Odds Ratio; ORa, Adjusted Odds Ratio; CI, Confidence Interval

**Table 4:** Unadjusted and adjusted analysis to assess adverse fetal and neonatal outcomes (Quantitative) associated with GWG

Outcomes	Gestacional weight gain (IOM-2019)			
	Below	Above	Below	Above
	β (IC95%)	β (IC95%)	β adj (IC95%)	β adj (IC95%)
Abdominal subcutaneous tissue thickness (TSA)	-0.02 (-0.10-0.06)	0.01 (-0.06-0.09)	-0.00 (-0.06-0.06)	0.05 (-0.00-0.11)
Arm subcutaneous tissue area (TSB)	-0.14 (-0.35-0.07)	0.06 (-0.15–0.27)	**-7.99 (-8.97–7.02)**	-0.08 (-0.17-0.01)
Thigh subcutaneous tissue área (TSC)	**-0.55 (-1.04 - -0.06)**	-0.16 (-0.62–0.31)	-0.25 (-0.45–0.05)	-0.01 (-0.20-0.19)
% Fat mass (%FM)	-0.28 (-0.76-0.20)	**2.52 (2.09–2.95)**	**-0.85 (-1.12–0.58)**	1.21 (0.94–1.48)
% Fat-free mass (%FFM)	1.51 (0.09–2.94)	0.87 (-0.41-2.15)	1.05 (-0.39-2.49)	0.52 (-0.86-1.91)
Birth length	**-0.79 (-1.07–0.51)**	-0.22 (-0.56-0.12)	**-0.76 (-1.03–0.49)**	-0.26 (-0.60-0.08)
Fetal weight	-62.9 (-136.4–10.6)	-39.8 (-112.1–32.5)	-17.73 (-38.07-2.61	7.50 (-13.03-28.04)
Birth weight	**-175.2 (-214.2–136.3)**	**98.5 (61.5-135.4)**	**-189.30 (-227.9–150.7)**	**90.14 (53.3–127)**

The association between each fetal or neonatal outcome and GWG. **Analyses adjusted for**: Maternal age; marital status; race/color; mothers’ education in years of study; BMI; gestational age in weeks; parity; number of rooms per person; lactose consumption; smoking; diabetes; serum levels of hemoglobin; glycated hemoglobin; cholesterol; triglycerides; HDL; LDL. β, beta coefficient of the generalized estimating equation. CI, confidence interval

## Discussion

In this study, we found that GWG outside the IOM recommendations is significantly associated with adverse outcomes, both fetal and neonatal. It was observed that a GWG below the recommendations increases the risk of IUGR, LBW, and prematurity. These findings align with the hypothesis that inadequate nutrition during gestation can compromise fetal development, which is corroborated by several studies [11–14]. On the other hand, a GWG above the recommendations was associated with an increased risk of LGA and changes in birth weight and length, suggesting that excess weight gain may predispose to obstetric complications and negatively impact the long-term health of the conceptus.

These findings corroborate previous studies that also found associations between GWG below and above the recommendations and adverse outcomes, indicating that inadequate GWG can have negative effects on the health of the fetus and newborn [14, 27–38].

Similar to our results, Goldstein et al. [28] in a systematic review and meta-analysis that investigated the association between GWG, and maternal and infant outcomes observed that inadequate GWG, whether below or above the IOM recommendations, is associated with adverse outcomes. Similar findings were reported in other studies [33, 35, 39]. The issue of prematurity deserves special attention, as women with premature babies have less time to gain weight during pregnancy, which may indicate reverse causality in the association between GWG and prematurity.

Liu et al. [30], in a retrospective cohort study with a sample of 9 million mother-child pairs to investigate the associations between GWG and adverse birth outcomes, showed that both insufficient and excessive GWG were associated with a higher risk of adverse outcomes, such as LBW, prematurity, and SGA. These findings highlight the importance of achieving an adequate balance in GWG, avoiding both restriction and excessive weight gain during gestation.

Furthermore, the relationship between GWG and adverse outcomes may be influenced by other factors, such as pre-gestational BMI. Studies by Chiavaroli et al. [32] and Liu et al. [30] reported that both maternal BMI and GWG are associated with adverse outcomes, suggesting that weight control before and during gestation is crucial. These findings are supported by other research, such as the study by Athukorala et al. [33], which found an increased risk of adverse outcomes in pregnant women with overweight or obesity, including prematurity and LBW.

However, it is important to note that GWG is just one of many factors that can influence birth weight. Other factors, such as maternal health, nutrition, smoking, and alcohol consumption, can also play a significant role. Additionally, the effect of GWG may vary in different populations, depending on factors such as BMI, age, ethnicity, parity, and underlying medical conditions of the mother [40].

Similar to our results, Goldstein et al. (2017b) in a systematic review and meta-analysis that investigated the association between GWG and maternal and infant outcomes observed that inadequate GWG, whether below or above the IOM recommendations, is associated with adverse outcomes. Similar findings were reported in other studies (Athukorala et al., 2010b; Chowdhury et al., 2022; Truong et al., 2015b). The issue of prematurity deserves special attention, as women with premature babies have less time to gain weight during pregnancy, which may indicate reverse causality in the association between GWG and prematurity.

Liu et al. (2022), in a retrospective cohort study with a sample of 9 million mother-child pairs to investigate the associations between GWG and adverse birth outcomes, showed that both insufficient and excessive GWG were associated with a higher risk of adverse outcomes, such as LBW, prematurity, and SGA. These findings highlight the importance of achieving an adequate balance in GWG, avoiding both restriction and excessive weight gain during gestation.

Furthermore, the relationship between GWG and adverse outcomes may be influenced by other factors, such as pre-gestational BMI. Studies by Chiavaroli et al. (2021) and Liu et al. (2022) reported that both maternal BMI and GWG are associated with adverse outcomes, suggesting that weight control before and during gestation is crucial. These findings are supported by other research, such as the study by Athukorala et al. (2010b), which found an increased risk of adverse outcomes in pregnant women with overweight or obesity, including prematurity and LBW.

However, it is important to note that GWG is just one of many factors that can influence birth weight. Other factors, such as maternal health, nutrition, smoking, and alcohol consumption, can also play a significant role. Additionally, the effect of GWG may vary in different populations, depending on factors such as BMI, age, ethnicity, parity, and underlying medical conditions of the mother [40],

In Brazil, a systematic review by Godoy et al. (2015) analyzed the recommendations for GWG and its impact on fetal outcomes. It was observed that both GWG below and above the recommendations are associated with adverse outcomes. Women with pre-gestational overweight or obesity have a higher risk of excessive gestational weight gain, which is associated with fetal macrosomia and high cesarean rates. Conversely, insufficient GWG can also lead to complications such as LBW and IUGR. In studies conducted in the Northeast and Southeast regions of Brazil, an association between macrosomia and women with pre-gestational overweight or obesity was observed [41–43]. In one of these studies, it was found that women with excessive GWG were more likely to have newborns with macrosomia [44]. Not only excessive weight and obesity influence perinatal outcomes, but insufficient GWG can also lead to complications, such as LBW and IUGR. Women with low pre-gestational body weight are more likely to have babies with LBW [45]. These findings highlight the importance of maintaining adequate GWG in Brazil to improve perinatal outcomes and reduce complications such as macrosomia and LBW. However, our study stands out by considering specific contextual variables of the Brazilian population, which may influence GWG and its outcomes. For example, the research by Drehmer et al. [46], in Brazil also noted the importance of the socioeconomic context in GWG and its effects on newborn health, but our study expands this understanding by including more detailed analyses of fetal and neonatal outcomes. Another point of distinction is our analysis of variables such as physical activity and smoking, which are known lifestyle factors that influence GWG. Studies like that of Nascimento et al. [47], in Brazil have shown that physical activity during pregnancy can help maintain GWG within the recommendations, which is consistent with our observations that maintaining adequate physical activity was low among women in the study.

Since GWG is a modifiable risk factor, it is possible to identify and correct its variations during gestation. Although various studies have demonstrated that appropriate interventions based on dietary changes can be effective in controlling weight gain and reducing the risk of complications during gestation [29], promoting healthy GWG can be an important element in avoiding adverse consequences for both the mother and the conceptus. Another important reference is the study by Dodd et al. [40], which conducted a systematic review of randomized clinical trials on antenatal interventions for overweight or obese pregnant women. The authors concluded that interventions such as dietary counseling and physical activity during gestation can result in modest reductions in maternal weight gain but did not have a consistent effect on the perinatal outcomes assessed. The target of the intervention should be women with abnormal weight, more prone to having adverse outcomes in future pregnancies. It is also concerning the increased risks of preterm birth, LBW, and IUGR in pregnant women with low weight, compared to those of normal weight. Even in the normal weight group, the risk of cesarean section, preterm birth, and excessive fetal growth increases with the elevation of BMI. Therefore, it is important to be concerned with women with overweight or obesity before gestation, but it is also necessary to strengthen the management of women with low weight and normal weight, who are often neglected. The findings of this study reinforce the need for clear and personalized guidelines for GWG, as well as the importance of regular weight monitoring during gestation. Nutritional and lifestyle interventions should be prioritized to help pregnant women achieve a healthy GWG.

In this study, we identified a notable and unprecedented finding: a significant association between insufficient GWG and reduction in the area of the fetal subcutaneous tissue of the arm but no reduction in the area of the subcutaneous tissue of the thigh or the thickness of the abdominal subcutaneous tissue, suggesting a regionalized response of fetal adipose tissue to maternal nutrition. This phenomenon may have important implications for neonatal health, as adipose tissue plays an essential role in energy reserves and thermoregulation of the newborn [48]. The uneven distribution of fetal adipose tissue may reflect an adaptation to a restrictive intrauterine environment, which may predispose the child to long-term metabolic and health challenges [49]. Furthermore, metabolic programming, which can be influenced by GWG, is a potential mechanism that may explain variations in the accumulation of fetal adipose tissue and its consequences for child health [50]. Maternal obesity and excessive GWG have been associated with epigenetic changes that may affect fetal development and the risk of metabolic diseases later in life [51]. On the other hand, prenatal exposure to insufficient GWG and very severe maternal obesity have also been associated with adverse neuropsychiatric outcomes in children, highlighting the importance of nutritional balance during gestation [52, 53].]

Limitations of this study include the lack of dietary data from pregnant women and the observational nature of the study, which prevents the inference of direct causal relationships. Additionally, despite adjustment for various covariates, we cannot rule out the possibility of residual bias. Future research should focus on better understanding the biological mechanisms linking GWG to adverse outcomes and on developing effective interventions to promote healthy GWG. Despite these limitations, the study has potentialities, such as being prospective and population-based, which allows the evaluation of a wide range of maternal, fetal, and neonatal outcomes. The prospective cohort approach and the use of objective measures, such as ultrasonography and air displacement plethysmography, contribute to obtaining accurate information about fetal development. The inclusion of various covariates related to socioeconomic, demographic, lifestyle, obstetric history, and clinical profile of pregnant women allows a comprehensive analysis of the factors associated with the outcomes evaluated.

Therefore, the need for more personalized GWG guidelines and the importance of targeted nutritional interventions during gestation are reinforced by these findings. Understanding the underlying mechanisms governing the distribution of fetal adipose tissue and its implications for child development and long-term health is crucial for optimizing perinatal outcomes and for the formulation of effective public health policies.

## Conclusion

This study underscores the imperative for targeted policies and interventions to manage GWG, which is critical for fetal and neonatal health. Effective weight monitoring and control strategies are essential to prevent insufficient or excessive gains, fostering a healthy pregnancy and averting neonatal complications. Access to information and support regarding appropriate GWG is crucial for maternal-infant well-being, and early intervention may forestall long-term health issues.

A novel finding of this investigation is the association between inadequate GWG and a reduction in the fetal subcutaneous arm tissue area, indicating a regionalized response of fetal adipose tissue to maternal nutrition. This could have significant implications for neonatal health and child development, suggesting that the distribution of fetal adipose tissue is a sensitive marker of the intrauterine environment. GWG guidelines should be tailored to reflect these insights, with a focus on personalized, evidence-based approaches. It is imperative that women achieve healthy weight targets during pregnancy, considering the regionalized response of fetal adipose tissue in the development of future nutritional guidelines and interventions.

### Electronic supplementary material

Below is the link to the electronic supplementary material.

Supplementary Material 1

## Data Availability

The datasets produced during this study will be accessible upon request directed to the corresponding author.
