# Supplementary material for: The impact of gestational weight gain on fetal and neonatal outcomes: the Araraquara Cohort Study
Source: BMC Pregnancy Childbirth. 2024 Apr 25;24:320. doi: 10.1186/s12884-024-06523-x (PMC11044382; doi:10.1186/s12884-024-06523-x)
Supplement: Supplementary file 1 — Supplementary Material 1 [file 12884_2024_6523_MOESM1_ESM.docx]

**Supplementary Material**

**Figure S1.** Theoretical model of the minimum set of adjustment covariates indicated by the DAG for the impact of Gestational Weight Gain on Fetal and Neonatal Outcomes.

The proposed model is represented by the DAG, demonstrating the relationships between variables associated with gestational weight gain, as well as fetal and neonatal outcomes. In the DAG, GWG is the exposure variable, that is, it is the variable that is intended to be investigated as influencing fetal and neonatal outcomes. From gestational weight gain, arrows are directed towards fetal and neonatal outcomes, indicating that gestational weight gain may have a direct impact on these outcomes.

**
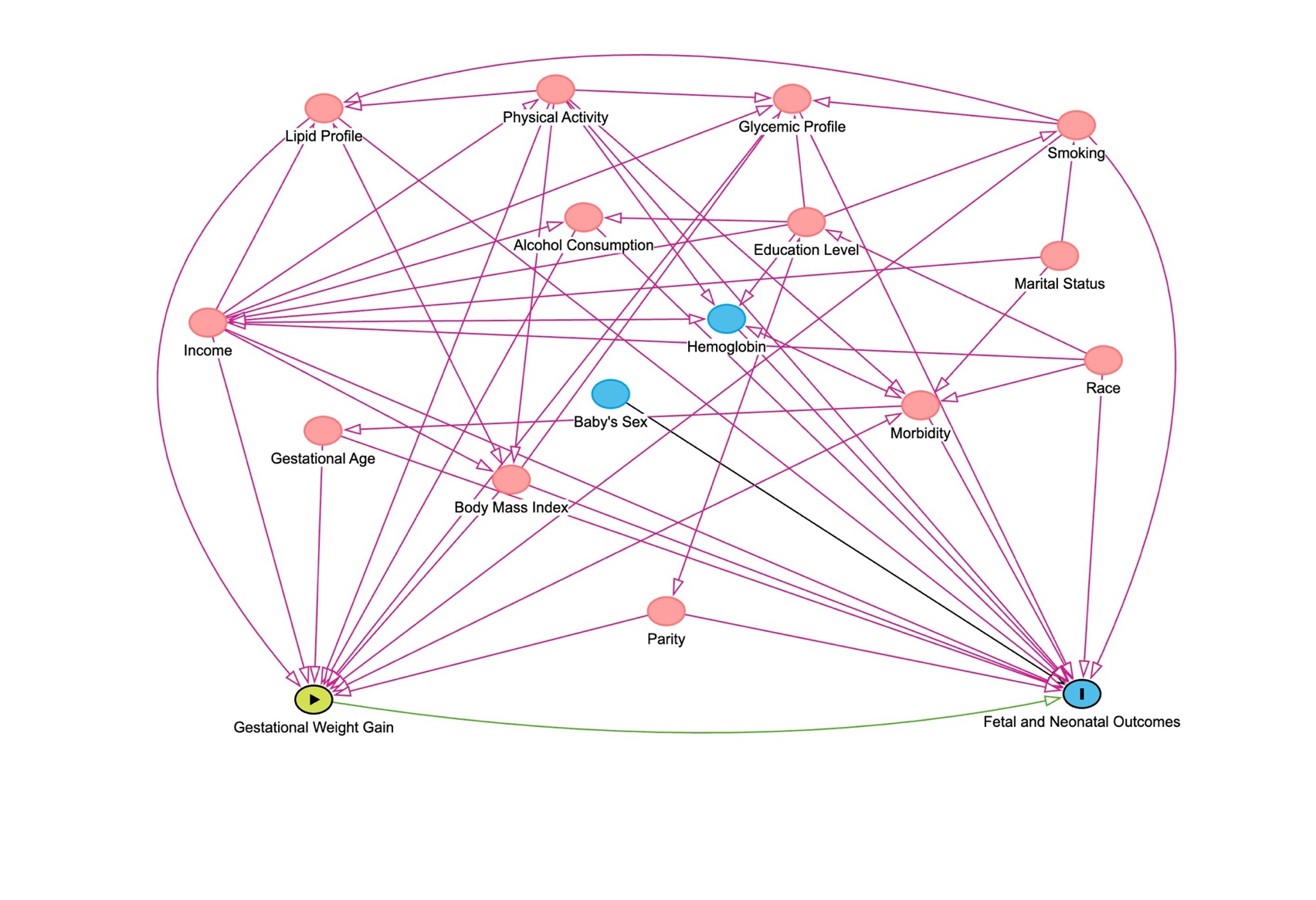
**
